# Intramedullary conus medullaris metastasis from prostate carcinoma: A case report and review of the literature

**DOI:** 10.3892/ol.2014.1808

**Published:** 2014-01-16

**Authors:** ZENGBAO WU, SIYI XU, CHUNLONG ZHONG, YANG GAO, QIANG LIU, YAN ZHENG, YANG GUO, YONG WANG, QIZHONG LUO, JIYAO JIANG

**Affiliations:** 1 Department of Neurosurgery, Renji Hospital, Shanghai Jiao Tong University School of Medicine, Shanghai 200127, P.R. China; 2 Department of Pathology, Renji Hospital, Shanghai Jiao Tong University School of Medicine, Shanghai 200127, P.R. China

**Keywords:** conus medullaris metastasis, prostate adenocarcinoma, intramedullary spinal cord metastases, prognosis, treatment

## Abstract

Intramedullary spinal cord metastases (ISCMs) are rare and account for 4–8.5% of central nervous system metastases. Only one case of biopsy-proven ISCM due to prostate cancer has previously been reported. The current study presents an additional unique case of a 74-year-old male who developed symptoms from an intramedullary conus medullaris metastasis as the first manifestation of prostate adenocarcinoma. To the best of our knowledge, this scenario is even more rare and has not previously been reported. The tumor was radically resected*,* followed by androgen blockade treatment. The patient’s neurological deficit significantly improved, with no tumor recurrence during the follow-up period. In addition, the present study provides an overview of the previous literature concerning ISCMs from prostate cancer, and discusses the treatment options.

## Introduction

Prostate adenocarcinoma is the second most common cause of cancer-related mortality in males, affecting up to 70% of males over the age of 80 years (1). In total, up to 10% of patients exhibit skull and spinal metastasis, while intramedullary spinal cord metastases (ISCMs) are rare and account for 4–8.5% of central nervous system metastases (2). Only one previous study of biopsy-proven ISCM due to prostate cancer has been reported (3). The current study presents an additional unique case of ISCM from prostate adenocarcinoma, in which the symptoms of conus medullaris dysfunction from the metastasis preceded the detection of the primary tumor. To the best of our knowledge, this is the first case in which conus medullaris dysfunction was the first symptom (4,5). This scenario is even more rare. The tumor was radically resected, followed by androgen blockade treatment. The patient’s neurological deficit significantly improved, with no tumor recurrence during the follow-up period. However, considering the rarity of ISCM, no previous controlled study has compared surgery, radiotherapy and androgen deprivation. The present study provides an overview of the previous literature concerning ISCMs from prostate cancer and discusses the treatment options.

## Case report

The patient was a 74-year-old male who complained of numbness and hypoesthesia in the lower limbs, together with back pain. The patient also experienced a loss of strength in the lower extremities. All these symptoms began four months prior to admission. Three weeks following the onset of these early-stage symptoms, the patient experienced difficulty in walking and a decrease in sensation from T12 to S5. Furthermore, the patient developed dysuria and worsening sphincter dysfunction. At the time of referral to the Department of Neurosurgery (Renji Hospital, Shanghai, China), the patient was no longer capable of walking alone. The patient provided written informed consent.

### Examination

On admission, the patient’s mental status, cranial nerve, upper extremities and general physical examination were normal. Neurological examination demonstrated that the legs were weak with hypoalgesia on both sides from T12 downwards. The Babinski reflex was not evoked on the patient’s feet.

### Neuroimaging

**Figure 1:**
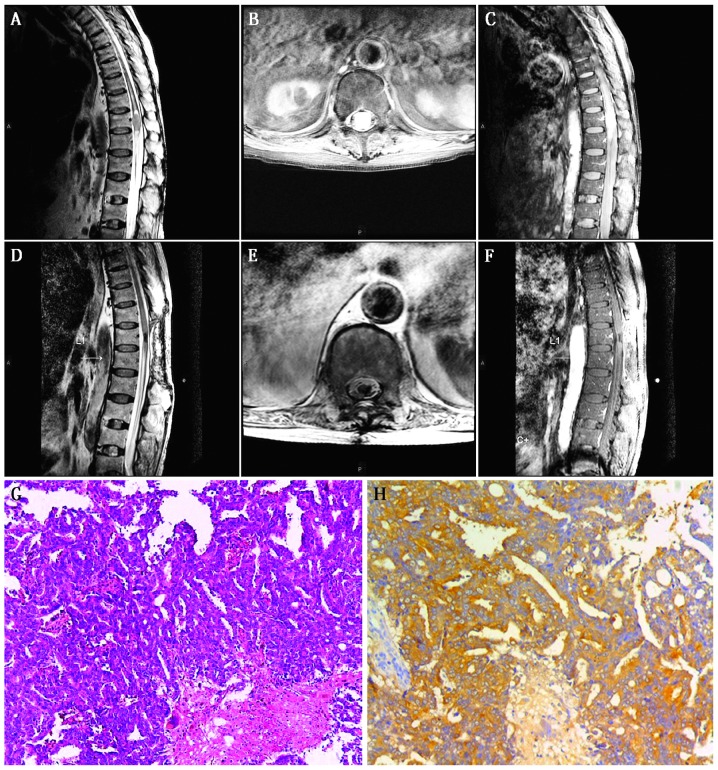
(A) T2-weighted sagittal and T1-weighted (B) axial and (C) sagittal gadolinium-enhanced MRI showing a ~15×28-mm spindle-shaped intramedullary mass located at the level of T12. (D) T2-weighted sagittal and T1-weighted (E) axial and (F) sagittal gadolinium-enhanced MRI demonstrating that the tumor tissue had been radically resected and a cavity had been formed. (G) Stained microscopic sections of the surgical specimen (hematoxylin and eosin; magnification, ×50). (H) Immunohistochemical staining revealed a positive reaction to prostatic acid phosphatase (magnification, ×100). MRI, magnetic resonance imaging.

Magnetic resonance imaging (MRI) revealed a spindle-shaped intramedullary lesion at the level of T12. A focal expansion of the spinal cord was identified with some surrounding edema. The lesion was ~15 mm in diameter and ~28 mm in the vertical dimension (Fig. 1A). Following administration of gadopentetate meglumine, the well-demarcated lesion was enhanced (Fig. 1B and C).

### Surgery

The differential diagnosis included ependymoma, astrocytoma or a metastasis from an additional primary tumor. Since the patient’s neurological deficit continued to develop following admission and the diagnosis was uncertain, a standard thoracic laminectomy from T12 to L1 and an excisional biopsy were performed, with somatosensory and motor-evoked potential monitoring. When the dura was opened, an enlarged abnormal conus medullaris was immediately identified covered by tortuous dilated blood vessels. An intraoperative frozen biopsy demonstrated adenocarcinoma. The abnormal tissue was radically resected without any damage to the adjacent spinal cord tissues. When the conus medullaris appeared adequately decompressed, an uneventful closure was performed.

### Pathological observations

**Figure 2:**
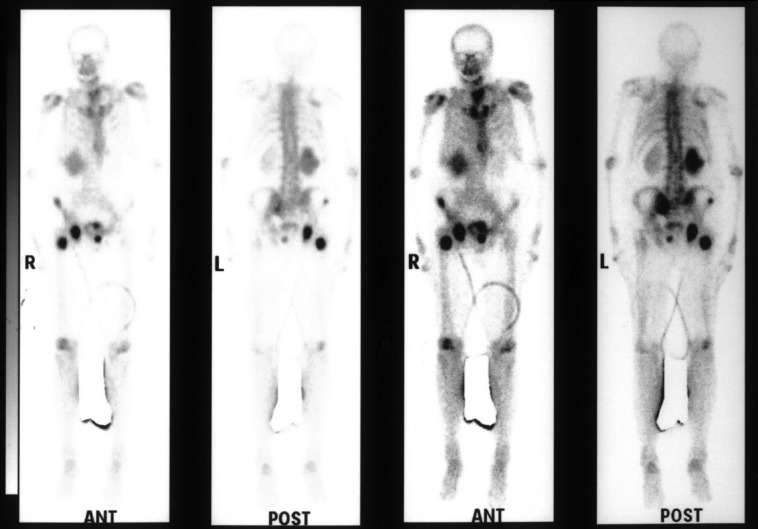
Single-photon emission computed tomography scan with ^99m^Tc-MDP demonstrated that the patient exhibited systemic bone metastasis involving the bilateral ilium, right acetabular, right pubis and right femoral.

Microscopic examination of the surgical specimen revealed sheets and nests of cells with abundant cytoplasm and prominent nucleoli (Fig. 1G). The immunohistochemical staining results were negative for transcription factor-1, CK7, CK20, SPA, GFAP, CA19-9, Muc1, Muc4, p53 and Vim; however, a positive reaction to prostatic acid phosphatase was observed (Fig. 1H). In combination with the microscopic characteristics, the pathology was consistent with metastatic adenocarcinoma of the prostate. Furthermore, the patient accepted a single-photon emission computed tomography scan, which showed multiple bone metastatic lesions (Fig. 2).

### Two weeks postoperatively

Postoperatively, the patient’s sensory disturbance did not improve significantly, but the motor examination marginally improved. In addition, the patient’s bowel and bladder function remained poor; therefore, a catheter was inserted. While the tumor was confirmed to be a metastasis of a prostate carcinoma, radiotherapy was suggested, but the patient refused due to the high cost. Subsequently, the patient received androgen blockade with leuprolide, flutamide and bicalutamide without any surgery on the prostate gland or primary tumor. No new neurological deficit was identified when the patient was discharged two weeks following surgery.

### Six months postoperatively

During the follow-up, androgen deprivation was continuously achieved with leuprolide, flutamide and bicalutamide. The prostate-specific antigen levels of the patient were almost normal, decreasing from an initial value of 22.25 to 0.47 μg/l. The patient’s bowel and bladder function improved significantly and the catheter was removed three months following surgery. Motor examination showed that the neurological deficit had also improved. A new MRI scan revealed that a cavity had formed (Fig. 1D–F) and, therefore, it was concluded that the tumor had been radically resected during surgery with no recurrence.

## Discussion

Tumor spread occurs primarily hematogenously via the arterial or venous system, mainly to the liver, lungs and bones. Secondly, dissemination occurs to the subarachnoidal space via the lymph vessels of the peripheral nerves. This route of spread is extremely likely when the cord is affected by leptomeningeal invasion (6). In ISCM patients, vertebral and other epidural metastasis is common, while metastatic intramedullary spinal cord tumors are rare. ISCM may be considered an infrequent observation at an advanced stage of the disease, usually accompanying the rapid progression of systemic cancer (2). Previously, Grem *et al* reported pain and weakness to be the most frequent symptoms (7). The authors reviewed 55 patients and found that bowel and bladder dysfunction were unusual early manifestations of intramedullary spinal cord metastasis, possibly as the time period from the onset of the neurological symptoms to the development of the full-blown neurological deficit is short. The incidences of the various primary tumors were as follows: Lung (small cell carcinoma in particular), 29–54%; breast, 11–14%; kidney, 6–9%; colorectal, 3–5%; melanoma, 6–9%; lymphoma, 4%; thyroid, 2%; and ovarian, 1%. In total, ~3% of cases are categorized as secondary tumors to unknown primary tumors (2).

Adenocarcinoma of the prostate affects up to 70% of males over 80 years of age and is the second most common cause of cancer-related mortality in males (3). Meningeal carcinomatosis, brain metastases and intradural extramedullary spinal lesions are uncommon. Only one previous case of biopsy-proven ISCM caused by adenocarcinoma of the prostate has been reported. However, the majority of cases of prostate adenocarcinoma exhibit no marked specific symptoms at the early stage. In the present case, it was confirmed that the patient had suffered from the tumor prior to the onset of conus medullaris dysfunction.

The median survival of patients with ISCM depends on several conditions. It is influenced by the nature of the primary cancer, the preoperative neurological status and the presence of systemic and/or CNS metastases; however, the differences are mostly without statistical significance (2). According to Schiff and O’Nill, the median survival is four months for patients receiving radiotherapy and two months for those not receiving radiotherapy (8). The authors’ observation is consistent with that of Grem *et al* who found that >80% of patients succumb to the disease within three months following the diagnosis of ISCM (7).

Currently, the surgical approach is more precise and less invasive and, thus, allows spinal cord tumor excision with an acceptable morbidity rate (2). The treatment of ISCM is mostly initiated to relieve pain and to preserve or stabilize neurological function; however, it does not extend the survival duration (5). In total, ~80% of patients succumb to ISCM within six months; therefore, a timely and effective microsurgery is feasible (1). The majority of prostate cancer tumor cells maintain androgen dependence. Hormone therapy is the current primary treatment for metastatic prostate carcinoma, which implies the lack of circulating testosterone to activate the androgen receptor (AR) in tumor cells, and it may be accomplished by a reduction in testosterone production or by AR blockade (9). However, considering the rarity of ISCM, no previous controlled study has compared surgery, radiotherapy and androgen deprivation.

In a previous study analyzing a unique adenocarcinoma in a prostate ISCM patient, the patient initially received a partial prostatectomy and androgen blockade and accepted decompression surgery for the ISCM. During the follow-up, the patient was treated with CyberKnife radiosurgery and neurological deficits remained (3). In the current case, the patient was unaware of the severity of his disease progression until the occurrence of dysuria and worsening sphincter dysfunction. This is the first case in which conus medullaris dysfunction was the first symptom onset. The patient refused to accept the radiotherapy due to the high cost and, therefore, only received curative resection of the metastatic tumor and hormone therapy for the prostate adenocarcinoma. The patient recovered well, and it appeared that radical surgery and effective androgen blockade may also treat the prostate adenocarcinoma in ICSM patients, particularly those with conus medullaris metastasis. Therefore, providing patients with successful palliation and improving their quality of life also requires multidisciplinary strategic treatment planning.
